# Perceived precarious life: a SEM model for re-dimensioning of precarious work and its impact on mental health

**DOI:** 10.3389/fpubh.2023.1254843

**Published:** 2024-01-05

**Authors:** José Antonio Llosa, Esteban Agulló-Tomás, Sara Menéndez-Espina, María Luz Rivero-Díaz

**Affiliations:** ^1^Department of Social Education, Faculty Padre Ossó, University of Oviedo, Oviedo, Spain; ^2^Department of Psychology, Faculty of Psychology, University of Oviedo, Oviedo, Spain

**Keywords:** precarious work, job insecurity, SEM, social exclusion, perceived precarious life

## Abstract

**Introduction:**

Precarious work is one of the most studied concepts related to work, and its effects have been analyzed in relation to variables such as mental health and wellbeing. However, there is a tendency to atomise the analysis of precarious work, without understanding that people's working life is intertwined with other areas of their life.

**Objective:**

Faced with this situation, this paper presents the concept of perceived precarious life, which is aligned with contemporary models of social inclusion and exclusion. Thus, perceived precarious life comprises variables of labor precariousness, social support and hopelessness in the family economic situation.

**Methods:**

To test this idea, a structural equation model (SEM) is presented, which tests the structure of the construct of perceived precarious life by relating it to mental health and coping strategies.

**Results:**

After testing the fit of the model in both men and women, a SEM path analysis is designed between the variables, observing that perceived precarious life has an effect on mental health (β = 0.635, *p* < 0.01). This relationship is mediated by unproductive coping strategies (β = 0.142, *p* < 0.01).

**Conclusion:**

This model exposes a broad and integrated conceptualization of precariousness, combining aspects of work, relationships and hopelessness, which allows for an understanding of the integral experience of precariousness.

## 1 Introduction

The perspective of symbolic interactionism allows for a conception of the subject in which the spheres of a person's life are interconnected with one another (1). Not in a sequence of causes and effects, but in an interrelated understanding. In this way, the theoretical model of social inclusion and social exclusion of Subirats (2) is a congruent conceptualization, since it states that the notion of social exclusion is found in the interaction between elements related to participation in the market (materialized in employment), the social sphere, and the spaces for citizen participation. This theoretical proposal has its origins in the approach of Castel (3) with similar epistemological and conceptual characteristics. What these conceptualisations propose is to focus on the interaction of elements in order to have a holistic vision of the condition of social exclusion, widely studied in sociology, social psychology, and social sciences as a whole (4).

With regard to the sphere of work, precarious work has been one of the most commonly studied areas of concern (5). In its theoretical conceptualisations it is presented as a set of non-standardized work circumstances (6, 7). That is, the conceptual complexity of precarious work invites us to analyse the phenomenon in terms of social constructionism (8, 9). It is necessary to determine, first, what are the culturally desirable working conditions, and, second, the construction of the idea of precarious work contextualized in these and defined on the basis of those situations that restrict the so-called standard situation.

Against this background, there have been attempts to characterize precarious work as “employment characterized by insecurity, low pay and limited social benefits” (10) or a process that includes instability, low protection, uncertainty, and economic or social vulnerability for Rodgers (7). In a broader perspective for the International Labour Organization, precarious work implies: “a means for employers to shift risks and responsibilities on to workers” (11). Finally, we find other more comprehensive approaches such as Benach et al.'s “multidimensional construct that includes dimensions such as job insecurity, individualized relations between workers and employers, low income, economic deprivation, limited rights over the organization of the workspace, as well as limited social protection” (12). Finally, Agulló-Tomás (13) points out the constant ambiguity in which the concept has evolved: “It will continue to be a concept tinged with ambiguity due to the fact that not all unstable jobs are precarious. But we can say that all precarious jobs are unstable” (13). All conceptualisations are constructed within the context of Western employment, which, while vastly heterogeneous, shares common basic elements in what we refer to as standardized. Some of these elements could be stability, the fact of maintaining a formal contractual relationship, a minimum income, or the possibility of working full-time if the employee wishes. The second important advance in the study of precarious work involves introducing a psychological perspective into the analysis. Not only understanding precariousness on the basis of objective characteristics derived from the job, but also the subjective perspective of this job (5, 10). This subjective experience has materialized in an equally broad conceptual corpus. Constructs such as quality of work life, work stress, burnout or job satisfaction have been gaining relevance in the scientific literature (14, 15). Probably, one of the most important and recent ones for the analysis of job insecurity from a psychosocial perspective is subjective job insecurity (16). This is defined as the fear of losing a job that one wishes to keep (17).

## 2 Model design

### 2.1 Conceptual model of precarious life as a broad proposal for understanding precariousness

Although precarious working conditions have been extensively linked to aspects of physical, social, and psychological wellbeing (18), they have also been understood as a fundamental element in relation to the analysis of social exclusion (19–21). For example, the United Nations Sustainable Development Goals (SDGs), in their search for more dignified living conditions, introduce decent work as the eighth goal (https://sdgs.un.org/). For its part, the European Union, in its current reference policy document for combating social exclusion (The European Pillar of Social Rights) (22), includes several principles related to the quality of employment.

However, the conceptual corpus of precarious work has followed a trend toward atomisation and specificity, and there are currently conceptual difficulties in understanding the interactive logic that precarious work has with other spheres of a person's life. In this sense, the group of women thinkers Precarias a la Deriva (23) proposes a model that understands precariousness without generating a duality between precarious work and other spheres of life: “we know that precariousness is not limited to the world of work. We prefer to define it as a juncture of material and symbolic conditions which determine an uncertainty with respect to the sustained access to the resources essential to the full development of one's life. This definition permits us to overcome the dichotomies of public/private and production/reproduction and to recognize the interconnections between the social and the economic”. That is, an understanding of precariousness that transcends labor, assuming other spheres of a person's life as interactive elements of the work experience, is presented. In order to make this theoretical model concrete, we propose a psychosocial approach, in what we call in this research work Perceived Precarious Life. This refers to a set of dimensions related to the person's experience of precariousness in the workplace and in other spheres of life in a unitary and integral manner. The scientific literature tells us that the development of work is bidirectionally linked to the relational plane: first, because employment, when it is of quality, allows the development of perceived social support (24). Second, because sources of social support are determinant elements for the possibility of finding and keeping quality jobs (25). On the other hand, hopelessness in the family economic situation is a second element intimately linked to employment relationships, and again in a bidirectional sense (26). Hopelessness in the family economic situation understood as the expectation of whether material conditions may improve in the future and the analysis of current material conditions in comparative terms. Thirdly, it is worth including aspects naturally linked to the labor market and the precarious work: the ability to control one's work, referring to high levels of autonomy, which is pointed out as an indicator of job quality; job satisfaction; and job insecurity, which is closely related to hopelessness (23).

These three elements: labor precariousness, perceived social support, and hopelessness in the family economic situation, are what are theoretically formulated as perceived precarious life. They provide a comprehensive theoretical framework for the concept of subjective precarious life, generating an interactive view of precariousness that is broader and more holistic, and also complementary, to that proposed by the existing analysis of precarious work. This prism assimilates the fact that precarious work is perceived and experienced in a holistic way. Likewise, the concept of perceived precarious life, if mathematically tested, allows for a psychosocial contribution to the interactive and interconnected model of social exclusion presented by Subirats (2) and Castel (3).

In addition, this model is based on the conception that work is a central element in industrial societies, impacting not only individuals' work spheres but also other aspects of their lives. Similarly, as a shared societal value that shapes the political and economic course of countries (27), it directly affects employed individuals as well as those who are unemployed or not actively working. Therefore, in industrial societies, the absence of decent work leads to situations of social exclusion and precarious lives. This idea is supported by works such as Standing (28), Agulló-Tomás (29), Lu et al. (30), Redmond et al. (31), and Halleröd et al. (32), among others.

### 2.2 Variables in the perceived precarious life model

#### 2.2.1 Precarious work in the model

With regard to precarious work, the subjective perspective of the concept is included. Starting from the conceptual elements of Rodgers (7) we will highlight three that make them concrete and will be included in the model of perceived precarious life: work control, work satisfaction and subjective job insecurity. With the work control variable, we can approach the ideas of social vulnerability from Rodgers model (7). Social vulnerability in the Rodgers model is closely linked to job fulfillment and the ability to have autonomy. Through the work satisfaction variable, we have a framework to consider the economic vulnerability of precarious employment, as well as aspects related to future prospects in work. Finally, job insecurity accounts for the dimension of uncertainty proposed by Rodgers (7) in his model. These are constructs of the subjective understanding of labor precariousness, relevant in a labor framework characterized by progressive deregulation (33). The current model of flexible employment implies a destabilization of formally stable contractual forms (34) on the one hand, and on the other, a loss of meaning for indefinite or permanent forms of employment. This has been observed in the prevalence of job insecurity among the population with stable employment contracts, when jobs are of poor quality (21).

The understanding of the current work control concept is articulated in Kasl's demand-control (job strain) model (35). What this model posits is that job control is understood in relation to the demands of the same (36). Thus, control over the job is summarized as the capacity for autonomy to plan a response to the demands of the job itself. It includes a competency perspective (perceived self-efficacy) contextualized in a particular work environment. When a worker exhibits low ability for control, it is understood as a deterioration of job quality, and results in a major stressor in the employment relationship. Thus, low work control has been related to variables directly related to work, such as low motivation or intention to leave the job, and also to variables external to the job, such as a decline in mental (37, 38) or physical (35) health. Specifically, in mental health, it has been observed in longitudinal approaches that a sustained increase in job control has a favorable impact on mental health (39). Furthermore, the conditions that pose the greatest risk to health are those that represent low work control, together with isolation (40). This last finding empirically evidences the psychosocial approach of the construct. Finally, the study of work control has also reflected a gender dimension: in a German sample of workers it was shown that depressive symptomatology linked to lack of work control in women was twice as high as the indicators recorded in the case of men (38).

Work satisfaction is related to perceived wellbeing in work contexts (41). It is a subjective experience or appraisal of the objective conditions of employment, which therefore has a psychosocial character. The assessment of job satisfaction does not depend exclusively on the employment conditions, but also on relational elements in the work context. In this construct, phenomena related to both external and internal conditions of the workplace are observed. The external factors are associated with objective aspects of the job, such as salary, and the satisfaction it generates for the worker. The internal factors are related to intrinsic aspects, such as the perception of opportunities for career development (42, 43). These two dimensions characterize work satisfaction as a complex set of variables that are explained based on the worker's experience in a specific work context (44). In the case of this research, we start from the approach formulated in the methodology of the European Working Conditions Survey 2010 (EWCS 2010), which measures this construct in different European contexts through Eurostat. When delving into the work satisfaction construct, has been observed that in people with diversity, tolerance in the workplace is a determining factor for work satisfaction (45). Job satisfaction, a construct widely analyzed in Work and Organizational Psychology, has been found to be related to a decrease in motivation (46); also that in positions of greater responsibility and decision-making capacity, work satisfaction tends to increase (47), as well as increasing with higher salaries or the possibility to reconcile work and personal life. In general terms, job satisfaction is related to mental health (mainly depression and anxiety), and is particularly so in the case of women (47). All these aspects relate work satisfaction to an indicator of labor precariousness that should be taken into consideration, associated with Rodgers (7) conceptualization of precarious work in the dimension of vulnerability.

In addition, the analysis by Cao et al. (48) which shows that work satisfaction is related not only to mental health, but also to social capital, is important. Social capital is related to Bourdieu's theory of habitus (49) and is, in psychology, a variable that analyses the social value that a person perceives about him/herself. It is a determining element to explore the situation of social inclusion.

Subjective job insecurity has been understood as a subjective approach to labor precariousness (50). Its interest grew in the context of the 2008 economic crisis, noting that in unstable employment settings its prevalence increases. Job insecurity specifically has been associated with a deterioration of general mental health, which has been observed in meta-analytic studies (5, 51, 52). It has also been found to be linked to suicidal ideation (53), as well as physical ailments. The latter, in particular, with disorders of a cardiovascular nature (54). This phenomenon also has a gender-differential approach: although the prevalence in men and women does not show significant differences, the determinants of job insecurity do differ. In the case of men, job insecurity is more closely related to the possibility of developing a successful career. In women, it was found to be more closely linked to the deterioration of material conditions implied by the possibility of job loss, as well as to the ability to reconcile work and family life (55).

#### 2.2.2 Perceived social support and precariousness

The second construct linked to the conceptualization proposed for perceived precarious life is perceived social support. A distinction is made between objective and subjective social support (56), the objective being the quality and quantity of support that the person has in their network, and the subjective being the perception of support in their environment. In conceptual congruence with the model of perceived precarious life, we will specifically analyse perceived social support. The conceptualization of perceived social support is summarized in the function that support fulfills. Thus, support exhibits instrumental functions, which authors such as Vaux (57) or Sherbourne and Stewart (58) understood as concrete and practical help, both financial and the provision of advice. On the other hand, perceived social support also fulfills what we call expressive functions. These are assimilated into affective and emotional functions (59). This construct has been related both to the situation of precariousness and social exclusion, as well as its effects on mental health. With regard to the work context, the absence of social support has been linked to workaholism or stress (60), and its presence to commitment and motivation in the company (59). On mental health, social support has been shown to be a protective factor that has been studied in multiple contexts: e.g., people in in-work poverty (21), people experiencing chronic illness (61) or the COVID-19 context (62). A cross-cutting variable in the analysis of social support is gender, noting that women tend to experience worse social support, this being a determining factor in the mental health consequences of employment (24).

However, social support and the work context establish a two-way network. On the one hand, employment can be a source of social support when it is a quality job (24). On the other, contexts of precarious work, such as in-work poverty, lead to a deterioration of the person's social support in general terms (63). Likewise, perceived social support experienced in contexts external to work has been shown to be a moderating factor for the negative consequences of precarious work (64). The quality and quantity of social contacts has also been found to be a determinant of career success (25). This conceptualization shows that support is a core element in understanding precarious work. And this interactive relationship implies that poor quality work implies a deterioration of social support; however, when perceived social support is developed in contexts external to work, other work-related factors have less negative impact.

#### 2.2.3 Hopelessness in the family economic situation and precariousness

**Figure 1:**
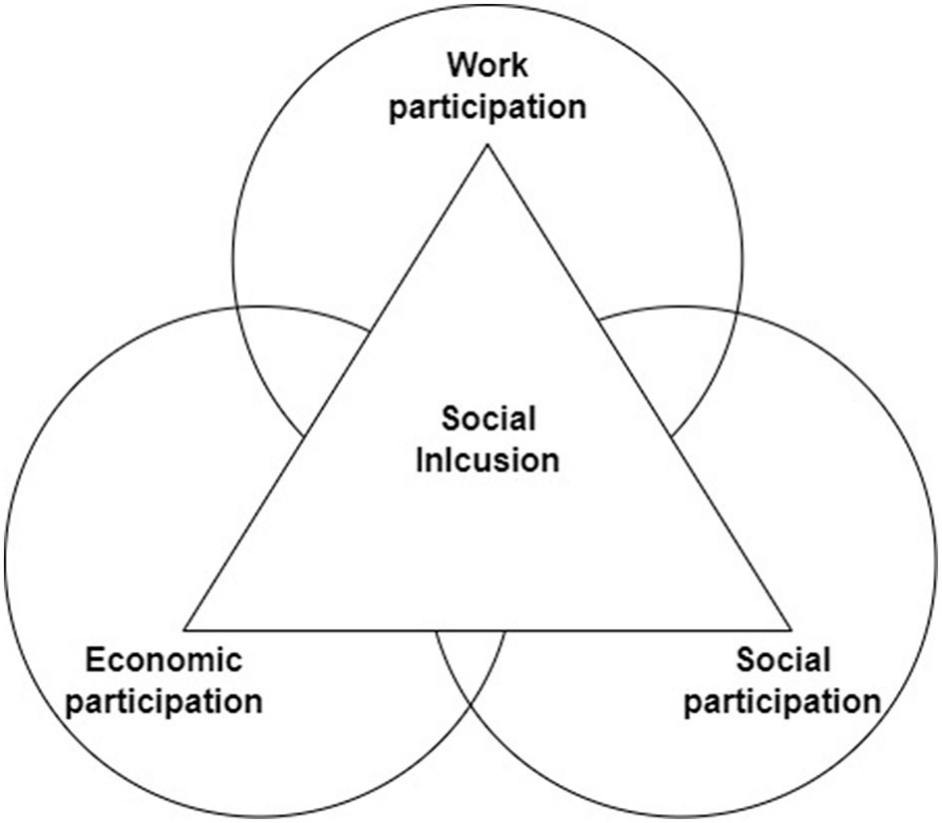
Subirats social inclusion/exclusion model. Interrelation between income and work activity (2).

The construct of hopelessness has been studied in Social Psychology in a traditional way, but its interest has grown considerably after COVID-19 (65–67). The study by Broos et al. (26) analyses the relationship between job stress and mental health symptomatology (depression and anxiety) at different points in time of the COVID-19 crisis, noting hopelessness as a moderating factor in this relationship. Furthermore, they conclude that not only work stress, but also the prospect of economic problems in the family were elements to be taken into consideration. The perspective we propose for the analysis of hopelessness in the perceived precarious life model assumes an uncertainty about the future, measured in terms of the family economy. For this reason, in the model, we have specified hopelessness specifically in the family economic situation. This refers to a low expectation of future income, as well as a limited positive evolution of the family economy. It is considered a different variable but related to the employment situation based on Subirats (2) model of social exclusion mentioned earlier. His model can be observed in more detail in Figure 1. The main source of income for a family comes from labor earnings, establishing this relationship at that point. However, in studies on labor precariousness, there is usually a focus on labor conditions, even on low wage incomes, but a more holistic perspective is not often considered, where labor incomes are evaluated in relation to the family's economic situation (68). Therefore, the construct of hopelessness in the family economic situation is proposed in this direction, since hopelessness is a construct of relevance in studies of poverty and social exclusion as a measure indicative of wellbeing (69, 70). Precarious work implies economic instability, which makes this association congruent.

### 2.3 Consequences and mediators in the pattern of perceived job insecurity: mental health and coping strategies

According to the interactive nature of the theorized model of perceived precarious life, it is to be expected that it presents an association with mental health. This conclusion is reached because it has been observed that the variables included in the model have a clear cross-sectional impact on the mental health status of the population. In the previous theoretical overview, we have seen the profound association of job insecurity, hopelessness in the family economic situation and a decrease in social support with a deterioration in mental health.

It has also been theoretically analyzed that coping strategies are an important variable both in contexts of precarious work and in contexts of exclusion. In particular, the finding that unproductive coping strategies are related to the mental health status of people in in-work poverty is relevant (63). Similarly, the moderation analysis developed by Menéndez-Espina et al. (24) shows that coping strategies play a moderating role between the condition of precarious work and mental health status. Likewise, it has also been observed that they play a role as mediators between different work-related stressors, including job insecurity (71), and their consequences on mental health (72, 73).

Lazarus and Folkman's theoretical approach conceptualizes coping strategies as “the constantly changing cognitive and behavioral efforts implemented to manage specific internal and external demands that are appraised as exceeding the resources of the person” (74). In turn Tobin et al. (75) divides the construct into productive and unproductive strategies. The former being those that are more beneficial for the individual, and the unproductive ones vice versa. In a sample-based study of in-work poverty, unproductive coping strategies (like self-criticism, social withdrawal, or wishful thinking) are analyzed, with a clear link to conditions of precariousness (21).

### 2.4 This study

**Figure 2:**
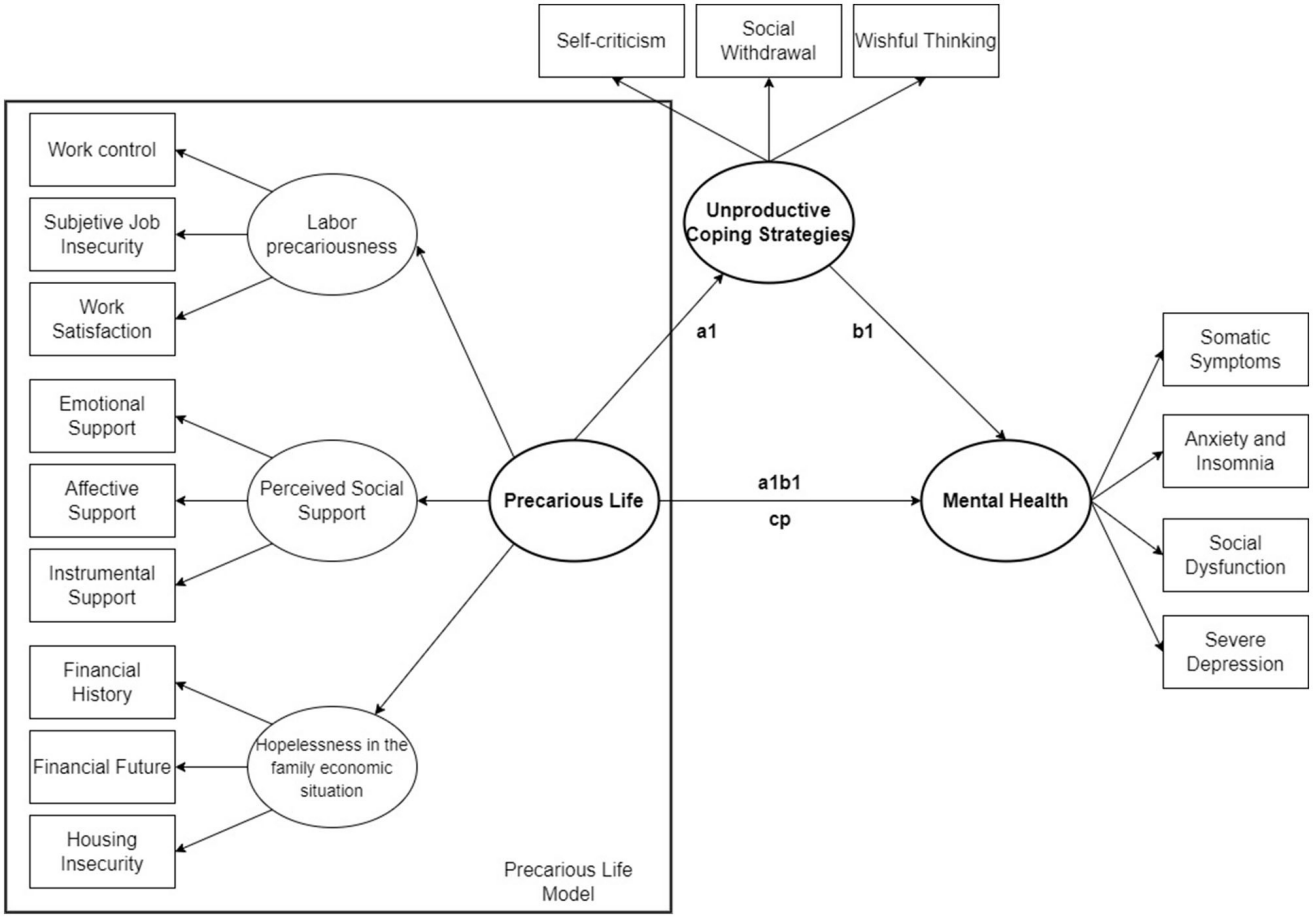
Hypothesized model: precarious life model with the variables of labor precariousness, social support, and hopelessness in the family economic situation, and its relationship in a mediation path model with mental health mediated by unproductive coping strategies.

With this theoretical proposal, the model presented in this research aims to test the construct of perceived precarious life. This is to be constructed containing a dimension of labor precariousness, a dimension of perceived social support and thirdly the concept of hopelessness in the family economic situation. To test this model, the intention is to assess its coherence by examining its relationship with other variables, as indicated in the rationale. The evidence encourages hypothesizing that perceived precarious life will be associated with mental health status, and that this relationship is mediated by unproductive coping strategies (Figure 2).

The research objective is to structure the concept of perceived precarious life as a holistic, integrated and interactive reconceptualization for studies on precarious work, and to test this construct in a model that relates it to mental health and coping strategies.

Hypothesis 1: The dimensions of labor precariousness, perceived social support and hopelessness in the family economic situation generate a congruent model for subjective precarious life in both men and women.
Hypothesis 2: Precarious life will have a mediated effect on mental health through unproductive coping strategies in both men and women.

## 3 Method

### 3.1 Participants

The inclusion criteria in the sample included in the study are limited to individuals who are employed in Spain. Secondly, exclusively the population who were formally employed at the time of responding. It only includes employees under a contract, self-employed individuals are not included in the study. Regarding age, data have been collected from individuals of legal age and up to the moment of retirement. The sampling method was a non-probabilistic convenience sampling method (76), through the self-administration of questionnaires in an online format via the Survey Monkey platform. The snowball sampling method was used, so that the sample progressively increased between the years 2021 and 2022. As a result of these criteria, a total of 2,054 people were studied, of whom 932 (45.4%) were men and 1,122 women (54.6%). The average age of the total sample was 36.63 years, with 35.68 for men and 37.41 for women. In terms of employment status, 942 people of the total sample had a permanent contract (50.8%) and 911 had a temporary contract (49.2%). These proportions are the same among men (428 with a permanent contract, 52% and 404 with a temporary contract, 48%) and women (504 with a permanent contract, 49.9% and 507 with a temporary contract, 50.1%).

### 3.2 Procedure

With a cross-sectional design, the set of questionnaires was applied in the following order: labor precariousness (perceived job insecurity, work control and work satisfaction); perceived social support (emotional support, affective support and instrumental support); the three variables of hopelessness in the family economic situation (financial history, financial future and housing insecurity); unproductive coping strategies (self-criticism; social withdrawal and wishful thinking); mental health (somatic symptoms, anxiety and insomnia, social dysfunction and severe depression), and sociodemographic data. The criteria for inclusion in the study were residence in Spain at the time of completing the questionnaire, formal employment, and age between 18 and 65 years. All persons who participated in the study did so voluntarily and were aware of the research objectives. Before starting to fill in the questionnaire, they gave their express consent to participate in the research process. This work had the support of the Ethical Committee of the Psychology Department of the University of Oviedo (Spain) and followed the principles for research with people contained in the Declaration of Helsinki of the World Medical Association (WMA).

### 3.3 Instruments

The presentation of measurement instruments used is grouped into the hypothesized variables that were subsequently tested:

#### 3.3.1 Labor precariousness

A set of questionnaires was used to measure Perceived job insecurity, Work control, and Work Satisfaction.

- Perceived job insecurity: perceived job insecurity was analyzed with the Job Insecurity Scale (JIS-8) validated in Spain (77) and originally developed by Pienaar et al. (78). It is an 8-item Likert-5 instrument, which provides a total score with a reliability α = 0.88. This scale shows higher rates of job insecurity as the total score increases.
- Work control: to study work control we used items from the European Working Conditions Survey 2010 (EWCS 2010). This set of 16 Likert-5 items is constructed by the statistical service of the European Union (Eurofound) and used in international data panels. Some items are, for example: “You are consulted before setting goals for your work” or “You have enough time to do your work”. In the sample used it yielded a reliability α = 0.83. For this measure, the higher the score on the scale, the less work control.
- Work Satisfaction: in the case of work satisfaction, a set of 6 Likert-5 items proposed in the European Working Conditions Survey 2010 (EWCS 2010) and developed by Eurofund was also applied. Example items in this scale are: “My job offers me good opportunities for career advancement” or “I receive a good salary for my work”. For the sample analyzed, it has an α = 0.71. In this scale, the higher the score, the lower the work satisfaction.

#### 3.3.2 Perceived social support

This was analyzed with the psychometric MOS scale, through the measurement of its three subscales: Emotional Support (10 Likert-5 items; α = 0.94); affective support (5 Likert-5 items; α = 0.86), and instrumental support (4 Likert-5 items; α = 0.87). The test was developed by Sherbourne and Stewart (58) and validated in the Spanish context (79). In these three measures, a higher score is indicative of greater support.

#### 3.3.3 Hopelessness in the family economic situation

To analyse hopelessness, three *ad-hoc* questions were designed, which we call Financial history (“When you compare your current household financial situation with that of 12 months ago, would you say it is better, worse or the same?”); Financial Future (“In relation to your household's financial situation in the next 12 months, do you expect it to be better, worse or the same?”), and Housing insecurity (“To what extent do you think it is likely or unlikely that you will have to leave your home in the next 6 months because you cannot afford it?”). Each of these items were used as variables independent of each other. In these three Likert response questions, the higher the score, the worse the situation the person experiences. Conceptually these three items are close to the theorisation of hopelessness in the family economic situation outlined in the introduction, looking at issues related to the person's expectations and trajectory, and focusing on the material conditions of life.

#### 3.3.4 Unproductive coping strategies

The set of subscales measuring unproductive coping strategies from the Coping Strategies Inventory by Tobin et al. (75), validated for the Spanish population (80). This inventory proposed a set of strategies classified as productive and unproductive. We took the unproductive ones, as they have been related to the conditions of precariousness (63). Specifically, these are Self-criticism (5 Likert-5 items, α = 0.94); Social Withdrawal (5 items, α = 0.81), and Wishful Thinking (5 Likert-5 items, α = 0.78). The authors of the scale opened up the possibility of using the subscales of the inventory independently. In this case, the higher the score on the scale, the greater the tendency to use these strategies.

#### 3.3.5 Mental health

It was studied with the General Health Questionnaire (GHQ) in its 28-item Likert-4 version (81) adapted to the Spanish population (82). The scores of the four subscales of the test were used: somatic symptoms (seven items), anxiety and insomnia (seven items), social dysfunction (seven items) and severe depression (seven items) that make up the model. In the Spanish validation, the authors reported that the scores have a reliability index above α = 0.90. In these subscales, the higher the score, the worse the mental health status.

#### 3.3.6 Sociodemographic data

The last part of the questionnaire asks about demographic aspects: gender, age, place of residence, employment status, and type of contract.

### 3.4 Data analysis

First, a descriptive and correlational analysis was carried out for the sample used, followed by a structural equation analysis. In order to respond to the hypotheses posed, a structural equation model (SEM) is designed according to the sequence of Singh and O'Brien (83). We started with a measurement model and then developed a SEM to analyse the relationship between the latent variables. The variables observed in the measurement model were structured into: labor precariousness (perceived job insecurity, work satisfaction and work control); perceived social support (emotional support, affective support and instrumental support); hopelessness in the family economic situation (financial history, financial future and housing insecurity); unproductive coping strategies (self-criticism, social withdrawal and wishful thinking), and mental health (somatic symptoms, anxiety and insomnia, social dysfunction and severe depression). Once the fit was tested, the structural equation model was carried out in order to observe the viability of the perceived precarious life construct, and to give it validity by relating it to the rest of the variables analyzed. It was hypothesized that the association between perceived precarious life and mental health is mediated by the unproductive coping strategies (Figure 1). Therefore, the total effect of the model was analyzed, as well as the direct effect of perceived precarious life on mental health, and whether this relationship is fully or partially mediated by unproductive coping strategies. The entire analytical process described above was conducted in the total sample and replicated in subsamples of men and women, carrying out a factorial invariance analysis between both groups using the Δ criterion in the fit indices proposed by Cheung and Rensvold (84).

These models have been developed with the Lavaan package v0.6-15 (85) in R v4.2.2 running on R Studio v 2022.07.2. Given the large sample size, Dampened weighted least squares (DWLS) has been used as the sample estimator, suitable for samples larger than 200 subjects (86). Analyses were carried out in all cases with 5,000 bootstrap samples. Chi-square and goodness-of-fit indices were used as fit indicators. We report goodness-of-fit indices common to models of a similar nature (87): Comparative Fit Index (CFI), Tucker-Lewis Index (TLI), Root-mean-square Error of Approximation (RMSEA) and Standardized Root Mean Squared Residual (SRMR). The interpretation criteria for these indices indicate a relative chi-square between 2.0 and 5.0. Regarding the criteria for goodness-of-fit indices: CFI ≥ 0.95; TLI ≥ 0.95; RMSEA ≤ 0.07; SRMR ≤ 0.08 (87). Having checked the fit of the model the mediating relationships between variables were examined (88).

## 4 Results

### 4.1 Measurement model

The measurement model for the total sample (Table 1) showed a significant chi-square [χ^2^ (95) = 418.328, *p* < 0.01], which would not support the model fit criterion. However, being a large sample size (*n* = 2,054), the literature indicates that it is usual to transgress this criterion, and the model fit is subject to the behavior of the other goodness-of-fit indices (89). Looking at the measurement model for the full sample CFI = 0.970, TLI = 0.962, as well as the residual indices (RMSEA = 0.041 CI 95% = 0.37; 0.045; SRMR = 0.044), the model fit was good. The reliability results for all latent variables have been above α > 0.70 in the measurement model, except for the latent variable hopelessness in the family economic situation. This is because this variable consists of *ad-hoc* items extracted from the Eurostat methodology that have not undergone a prior psychometric validation process. However, the model is conceptually coherent and demonstrates a completely adequate fit index. When studying the fit in the disaggregated samples of men and women, similar indices were found. Somewhat higher than the total sample for women with an increase in CFI of 0.15 and TLI of 0.19, and somewhat lower for men with a negative variation in CFI of 0.08 and TLI of 0.10. In any case, in all three models (total sample, men and women) the fit met the criterion.

**Table 1:** Measurement model adjustment.

	**Chi-square (df)**	**CFI**	**TLI**	**RMSEA (CI)**	**SRMR**
Total sample	418.328 (95)^**^	0.970	0.962	0.041 (0.037; 0.045)	0.044
Men	267.245 (95)^**^	0.962	0.952	0.045 (0.039; 0.051)	0.053
Women	186.049 (95)^**^	0.985	0.981	0.030 (0.023; 0.036)	0.040
Criteria value		≥0.95	≥0.95	≤ 0.07	< 0.08

^**^p < 0.01. Latent (and observed variables) in the model: labor precariousness (work control; perceived job insecurity, and work satisfaction), social support (emotional support; affective support and instrumental support) and hopelessness in the family economic situation (financial history; financial future and housing insecurity); unproductive coping strategies (self-criticism, social withdrawal and wishful thinking), and mental health (somatic symptoms, anxiety and insomnia, social dysfunction and severe depression).

The invariance of the measurement model between men and women was analyzed: both configural and metric, as well as scalar invariance, were assessed with the criteria ΔCFI < 0.01, ΔTLI < 0.01, ΔRSMEA < 0.015, and ΔSRMR < 0.015 for large samples (72). Configural and metric factorial invariance between groups (men and women) is achieved (Table 2). This indicates that the structure of latent variables in the designed measurement model is common between men and women, making a separate observational analysis with the model appropriate (78). However, scalar invariance, which would allow for *t*-test comparisons between models for men and women, is only partially achieved. It is necessary to block the exogenous variable wishful thinking for this purpose (79). For this reason, the gender analysis proposed by the results of our work with the path model will only be conducted in observational terms.

**Table 2:** Invariance multigroup test (women/men).

	**CFI**	**TLI**	**RMSEA**	**SRMR**	**Δ CFI**	**Δ TLI**	**Δ RMSEA**	**Δ SRMR**
Baseline model	0.970	0.960	0.041	0.044				
Configural	0.960	0.952	0.046	0.051	0.010	0.008	0.005	−0.007
Metric	0.957	0.950	0.047	0.053	0.003	0.002	−0.001	−0.002
Scalar	0.945	0.940	0.052	0.057	0.012	0.010	−0.005	−0.004
Scalar partial	0.950	0.946	0.049	0.055	0.007	0.004	−0.002	−0.002

### 4.2 Structural model

The fit of the structural model is also disaggregated for the total sample, as well as for men and women (Table 3). In the total sample the model fit yielded a chi-square χ^2^ (99) = 515.61, *p* < 0.001; CIF = 0.962; TLI = 0.953; RMSEA = 0.046 CI 95% (0.042; 0.050), and SRMR = 0.049. With the criteria followed in the measurement model we can conclude, also in this case, a good fit. Examining the case of men and women, a better fit was again observed in the subsample of women. In the case of women the CFI increases by 0.09 with respect to the total sample, and the TLI 0.12. In the case of men there was a negative variation with respect to the joint population model: 0.04 in the case of both CFI and TLI. The fit in the model tested in the female subsample was perfect, while in the case of males it is adequate in CFI and the residuals indices (RMSEA and SRMR), and almost perfect for the TLI (TLI = 0.949). One must conclude a correct fit of the structural model in the three sample sets analyzed.

**Table 3:** SEM model adjustment.

	**Chi-square (df)**	**CFI**	**TLI**	**RMSEA (CI)**	**SRMR**
Total sample	515.61 (99)^**^	0.962	0.953	0.046 (0.042; 0.050)	0.049
Men	287.92 (99)^**^	0.958	0.949	0.046 (0.040; 0.052)	0.055
Women	274.535 (99)^**^	0.971	0.965	0.040 (0.035; 0.046)	0.049
Criteria value		≥0.95	≥0.95	≤ 0.07	< 0.08

^**^p < 0.01.

**Figure 3:**
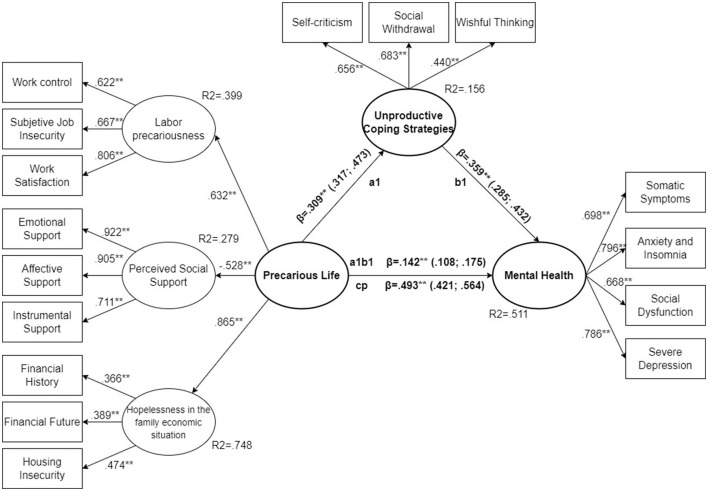
SEM structural model, total sample. **p < 0.01.

**Figure 4:**
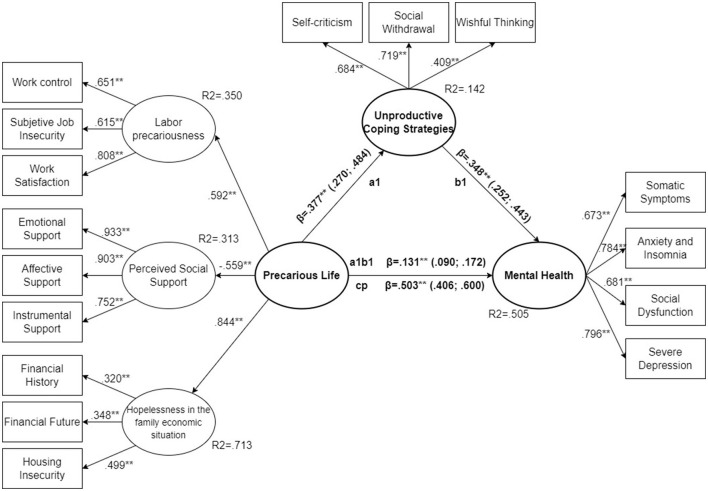
SEM structural model, women sub-sample. **p < 0.01.

**Figure 5:**
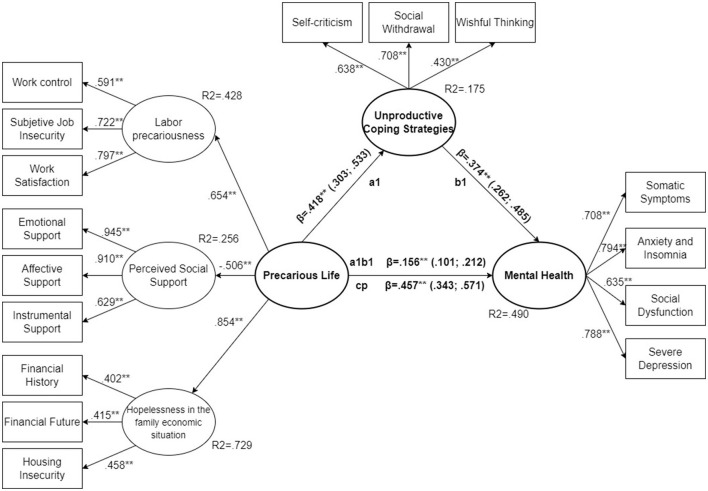
SEM structural model, men sub-sample. **p < 0.01.

All standardized parameters for the total sample (Figure 3) were significant (*p* < 0.01). They were above 0.4 for all variables included, except for two of the variables related to hopelessness in the family economic situation (financial history = 0.366, *p* < 0.001 and financial future = 0.389, *p* < 0.001). However, the parameter estimate is close to 0.4, and the *R*^2^ of this latent variable is high (*R*^2^ = 0.748). There was no clear imbalance between the parameter estimates for the three observed variables linked to the latent variable hopelessness in the family economic situation. The same situation was observed for women (Figure 4), where the observed variables associated with latent hopelessness (*R*^2^ = 0.713) showed statistically significant parameters of financial history = 0.320, financial future = 0.348 and housing insecurity = 0.499. Again, all model parameters were statistically significant (*p* < 0.001). In the case of men all estimators were above 0.4 (Figure 5) and were statistically significant (*p* < 0.001).

### 4.3 Hypothesis 1: perceived precarious life model

Hypothesis 1 tested the structure of the theoretical construct of precarious life in the total sample, as well as in men and women. The fit indicators of the models showed that the theoretical structure was adequate, following the initial theoretical model of Precarias a la Deriva (23) in all cases. The mediation analysis showed a significant total effect for the model (β = 0.635, CI 95% = 0.575; 0.694, *p* < 0.001) (Table 4). When replicated in women the total effect is also significant (β = 0.634, CI 95% = 0.550; 0.718, *p* < 0.001), as in the subsample of men (β = 0.613, CI 95% = 0.522; 0.703, *p* < 0.001) (Table 4). Thus, the association between perceived precarious life and mental health is observed, reaching not only the structural congruence of the perceived precarious life construct, but also providing it with validity.

**Table 4:** Indirect, direct and total effects of path SEM model total sample.

**Total sample**		**Est. standard**	**SE**	***p***
Indirect effects: perceived precarious life → unproductive coping strategies → mental health	a1b1	0.142	0.017	0.000
Direct effect: perceived precarious life → mental health	cp	0.493	0.037	0.000
Perceived precarious life → improductive coping strategies	a1	0.309	0.042	0.000
Improductive coping strategies → mental health	b1	0.322	0.039	0.000
Total effect	a1b1 + cp	0.635	0.030	0.000
**Men**				
Indirect effects: perceived precarious life → unproductive coping strategies → mental health	a1b1	0.156	0.028	0.000
Direct effect: perceived precarious life → mental health	cp	0.475	0.058	0.000
Perceived precarious life → improductive coping strategies	a1	0.418	0.058	0.000
Improductive coping strategies → mental health	b1	0.374	0.057	0.000
Total effect	a1b1 + cp	0.613	0.046	0.000
**Women**				
Indirect effects: perceived precarious life → unproductive coping strategies → mental health	a1b1	0.131	0.021	0.000
Direct effect: perceived precarious life → mental health	cp	0.503	0.049	0.000
Perceived precarious life → improductive coping strategies	a1	0.377	0.055	0.000
Improductive coping strategies → mental health	b1	0.348	0.049	0.000
Total effect	a1b1 + cp	0.634	0.043	0.000

If we analyse the standardized parameters of the variables that make up the construct of perceived precarious life in their relationship with mental health and unproductive coping strategies, we observe that all of them are significant (*p* < 0.001) both in the total sample and in the subset of men and women. Analyzing the total sample, the one with the strongest relationship was hopelessness in the family economic situation (0.865, *p* < 0.001). The association of the precarious life construct with labor precariousness (0.632, *p* < 0.001) and perceived social support (−0.528, *p* < 0.001) was similar, with the estimator of social support being negative. The scale used to detect the three social support variables (emotional support, affective support and instrumental support) indicates that the higher the score, the greater the social support (79). The model indicated that social support represents a relevant variable in the construction of perceived precarious life, but resulted in a protective factor: the higher the social support, the lower the precarious life. In the case of women, the relationship coincides (Figure 4) and also among men (Figure 5).

### 4.4 Hypothesis 2: precarious life mediated effect on mental health in both men and women

The second hypothesis tested the mediation of coping strategies on the effect between perceived precarious life and mental health. For all three samples the indirect effect (a1b1) was statistically significant (*p* < 0.01), but also the direct effect (cp) (*p* < 0.001). This implies that we are dealing with a partially mediated model.

For the total sample (Figure 3) the direct effect (cp) (β = 0.493, CI 95% = 0.421; 0.564, *p* < 0.001) accounts for 77.64% of the total effect, with 22.36% due to the mediating effect of unproductive coping strategies (β = 0.142, CI 95% = 0.108; 0.175, *p* < 0.001). The regression between perceived precarious life and unproductive coping strategies (a1) is significant (β = 0.309, CI 95% = 0.317; 0.473, *p* < 0.001), with an *R*^2^ = 0.156; as is the regression between unproductive coping strategies and mental health (β = 0.359, CI 95% = 0.285; 0.432, *p* < 0.001). The model explained an *R*^2^ = 0.511 for the mental health variable.

In the sample analyzed, comparing the direct effect of perceived precarious life between men and women, it is observed that in the case of men it accounts for 82.6% of the total effect (β = 0.457, CI 95% = 0.343; 0.571, *p* < 0.001) (Figure 4), while in the case of women it accounts for 74.55% (β = 0.503, CI 95% = 0.406; 0.600, *p* < 0.001) (Figure 5). This showed the mediating role of unproductive coping strategies; although significant in both cases, the female model was more relevant.

## 5 Discussion

### 5.1 Hypothesis 1: perceived precarious life model

The first hypothesis of this model presented the possibility of generating a congruent model of the perceived precarious life concept. This model could include labor precariousness, perceived social support and hopelessness in the family economic situation as dimensions. According to the results of the measurement model and the structural model of the SEM it can be stated that the hypothesis is fulfilled. Following the initial theoretical approach (23), the model of precarious work can be viewed in a broader, interconnected conceptual scope. Ultimately, this model of perceived precarious life allows for a holistic understanding of precariousness that includes and transcends strictly work-related precariousness. This is important, as the theoretical and empirical starting point is the fact that precariousness at work affects the life of the individual in a holistic way (2, 23). Although analytically relevant, it is conceptually risky to try to understand the effects of precarious work solely from the perspective of working conditions, due to the limitation of isolating the employment sphere of a person's life from all other aspects of their life (90).

Thus, this research generates a congruent model together with perceived social support and hopelessness in the family economic situation. Moreover, analyzing the estimators of the model, it is observed that precarious work is not the most relevant variable of the three. The model is balanced, which brings coherence to the proposal. In the sample analyzed, hopeslessness represents the variable with the greatest weight both in the total analysis (0.865, *p* < 0.001) and in the subsample of men (0.854, *p* < 0.001) and women (0.844, *p* < 0.001). In the interactive view put forward as a theoretical model, the need to understand precariousness in conjunction with its material effects was pointed out. Hopelessness in the family economic situation in the model takes the form of perceived economic security and housing security. Obviously, material possibilities are interconnected with the employment situation (19, 20), but both levels have to be looked at together.

Studies on precarious work have highlighted the effect of working conditions on mental health (12, 24). But it is less frequent that they consider the material or economic situation of the individuals and families analyzed. Given this, there is a possibility that the results of the studies may confuse precariousness and economic situation by not taking into account this relationship (21). Studies on in-work poverty have very often pointed to this type of link, as it is a situation that intersects precarious work and financial poverty. For example, it has been observed that workers who are in precarious work, and also poor, have consistently lower future prospects and social support (91, 92). Even lower than that of precarious workers who are not in poverty (21). It has also been observed that there is a gender gap in in-work poverty, as women tend to occupy not only more precarious jobs, but also lower paid jobs. The perceived precarious life model presented in the results allows us to discriminate material expectations from precarious conditions, and also incorporates the perceived social support variable.

The perceived social support variable, in the model, has a standardized parameter with a negative sign (−0.528, *p* < 0.001), indicating that it is a protective factor. At the same time, it is significant, which shows its relevance in the model designed. Social support is a protective factor for mental health (62), however, the absence of support points to a risk factor (63, 64). It is relevant to take this dimension into account in the model, because it has been observed that people in poverty (93), or those in precarious work (94), have fewer sources of support. However, it has also been found that sources of social support are a useful element in the wellbeing of this population (59), or if we talk about precarious work, closely related to the possibility to access employment (25).

The first hypothesis also tested the feasibility of the model among men and women. The results show that the structural model adjusts for both genders. The conditions of precariousness and social exclusion in population terms affect women more than men (95, 96). Phenomena such as the wage gap, the glass ceiling or the feminisation of poverty have been widely studied (97). What the perceived precarious life model brings to the discussion is evidence of a common structure for the construct between men and women. However, in the model's approximation to the social support variables, it is observed in the standardized parameters that among women the instrumental support variable has a greater weight (0.752, *p* < 0.001) than among men (0.629, *p* < 0.01). This variable is related to the fact of having direct help, and has been strongly related to caregiving tasks (55, 79). Previous research shows that women's experience of precariousness is much more closely linked to family support, because in current social models there is still a link between women and the demand for care (55). For this reason, one of the aspects of women's precarious work refers to work/life balance (23). This dimension is not explicitly taken into account in the perceived precarious life model designed, but it is possible to establish this relationship with social support.

### 5.2 Hypothesis 2: precarious life mediated effect on mental health in both men and women

The second hypothesis states that perceived precarious life is associated with mental health, and that this association is mediated by unproductive coping strategies. This hypothesis is partially fulfilled: indeed, precarious life, in the mediation model, has a significant overall effect on mental health (β = 0.635, CI 95% = 0.575; 0.694, *p* < 0.001). However, the mediation of coping strategies is not total, but partial. Thus, it should be taken into consideration that unproductive coping strategies are variables that are mediating between precarious life and mental health, but there would be other mediating or moderating variables in this relationship that we do not know of at the moment. Again, the partial mediation model is also present in the subsamples of men and women.

These results also validate the perceived precarious life model. The scientific literature reiterates that the variables that make up precarious life (precarious work, social support and hopelessness in the family economic situation) are related to mental health (12, 26, 60). It was therefore to be expected that the relationship would be found between the perceived precarious life construct as well. The same is true for coping strategies (24).

In addition to providing validity to the model, the results have interventional implications. Perceived precarious life is related to unproductive coping strategies, and these in turn to mental health. This relationship has also been found to be linked to precarious work conditions (98), and also with social exclusion (99). Unproductive strategies lead to a deterioration of mental health (63). Interventional impact relates to the ability to intervene on coping strategies (100). The coping strategies model of Tobin et al. (75) distinguishes between productive and unproductive coping strategies, whereby developing productive coping strategies, as a substitute for unproductive ones, may have a buffering role for the effects of precarious life on mental health.

This phenomenon also has a gender dimension, since, although it is a partially mediated relationship observed in men and women, in the case of women it has a greater indirect effect and is therefore more relevant. Therefore, in situations of perceived precarious life, there would be a greater tendency among women to implement unproductive coping strategies. Heras Recuerdo and Osca Segovia (101) showed in a study on the mediating role of coping strategies in the consequences of burnout that women have a greater tendency to use coping strategies, especially cognitive ones. It is therefore congruent that in the perceived precarious life model they have more of an indirect effect for women. On the other hand, men seem to use more behavioral coping strategies (102), which are not analyzed in our model.

However, the fact that mediation is partial and not total in all cases (total sample, and sub-samples of men and women) implies that intervention on unproductive strategies is not enough. There is therefore a dimension of intervention on public policies to prevent precarious life (103). Similarly, strengthening community models is an appropriate intervention strategy to generate social support processes among citizens in neoliberal contexts (104).

### 5.3 Limitations and future research

The model presented is constructed with a cross-sectional design, such that this model of perceived precarious life could be tested in longitudinal studies. It will make it possible to present a more robust model, and to control for macro-social variables that could be involved in different models. In addition, the hopelessness in the family economic situation latent variable is constructed with three items that address three variables (financial history, financial future, and housing insecurity). These three variables present a perspective of hopelessness in the family economic situation with a double purpose: an approximation perceived by the person and linked to expectations of a material nature. However, it would be important to have a psychometric scale that would allow a more robust approach to this measure of hopelessness. The selected sample is non-probabilistic. Despite including a large number of participants, not using a probabilistically selected sample implies a limitation in the generalizability of results (105). Future research that tests this model would be advisable to be conducted not only with longitudinal designs but also with probabilistic sampling. Lastly, in this regard, the study has the limitation of not including self-employed individuals in the labor force. This limitation is specified because the employment characteristics of the self-employed population are specific and therefore not easily comparable to those of individuals employed under a contract.

On the other hand, the designed measurement model achieves partial scalar invariance in the multigroup analysis between men and women. This represents a limitation, as comparison through *t*-tests or similar tests between men and women is not appropriate. Hence, we restrict ourselves to an observational comparison in this case.

Other limitation is that the model concludes in a partial mediation of coping strategies. It would be important to hypothesize what other variables may be found to be involved in the association of perceived precarious life and mental health, and to test them. Furthermore, it would be interesting to include coping strategies not only of a cognitive nature, which are those proposed by the model of Tobin et al. (75) used, but also strategies of a behavioral nature.

In any case, and presenting future research, the contribution of this work is the modeling of a broad construct to analyse perceived precarious life, which allows for a comprehensive analysis of the condition of precariousness. This means a new contribution in this field of specialization, offering a new conceptual, methodological and theoretical model, which will allow new possibilities for intervention, analysis and development in this field. It presents a new plane of development for the literature in this context, which can be exploited from various disciplines, with a very broad set of variables that could potentially be consequences of this phenomenon.

## Data availability statement

The raw data supporting the conclusions of this article will be made available by the authors, without undue reservation.

## Ethics statement

The studies involving humans were approved by Psychology Department, University of Oviedo, Spain. The studies were conducted in accordance with the local legislation and institutional requirements. The participants provided their written informed consent to participate in this study.

## Author contributions

JL: Conceptualization, Data curation, Formal analysis, Investigation, Methodology, Validation, Writing – original draft. EA-T: Conceptualization, Investigation, Supervision, Validation, Writing – review & editing. SM-E: Data curation, Formal analysis, Investigation, Methodology, Validation, Writing – review & editing. MR-D: Data curation, Formal analysis, Methodology, Validation, Writing – review & editing.
